# Can early switch to rituximab-bendamustine in a patient with follicular non-Hodgkin lymphoma progressing during R-CHOP be considered frontline treatment? A case report

**DOI:** 10.1097/MD.0000000000021440

**Published:** 2020-08-14

**Authors:** Claudio Cerchione, Davide Nappi, Gerardo Musuraca, Alessandro Lucchesi, Ilaria Cimmino, Fabrizio Pane, Amalia De Renzo, Giovanni Martinelli

**Affiliations:** a Hematology Unit, Istituto Scientifico Romagnolo per lo Studio e la Cura dei Tumori (IRST) IRCCS, Meldola; b Department of Hematology and CBMT, Ospedale di Bolzano, Bolzano; c Department of Translational Medicine, Federico II University of Naples; d Hematology Unit, Department of Clinical Medicine and Surgery, University Federico II, Naples, Italy.

**Keywords:** bendamustine, early switch, non-Hodgkin lymphoma, pegfilgrastim, R-CHOP, rituximab

## Abstract

**Rationale:**

Follicular non-Hodgkin lymphoma (fNHL) is a neoplasm characterized by an indolent course and chemosensitivity, but also by disease recurrence. Bendamustine is often used as frontline treatment or second line.

**Heading diagnosis:**

fNHL.

**Patient concerns:**

A 63-year-old Caucasian male with diagnosis of fNHL lymphoma underwent to cyclophosphamide, doxorubicin, vincristine, and prednisone associated with rituximab chemoimmunotherapy, during which interim reevaluation showed progressive disease and severe toxicity.

**Interventions:**

Early switch to rituximab-bendamustine.

**Outcomes:**

This regimen was well tolerated, patient compliance was optimal, there were no delays in administration and no infectious episodes. An interim reevaluation after 3 courses revealed that the patient was fit, the blood cell count was normal, and lymphadenopathies and nocturnal sweating had completely regressed. Of note, the PET/CT scan did not show fluorodeoxyglucose pathological uptake, clearly confirming disease regression.

**Lessons:**

Early switching to a bendamustine-rituximab-based scheme, even during conventional chemotherapy, decreases toxicity and reduces the risk of treatment interruption or delay, with favorable effects on overall response and prognosis.

## 1 Introduction

Follicular non-Hodgkin lymphoma (fNHL) is the most common type of non-Hodgkin lymphoma in the United States and Europe. It is a heterogeneous group of indolent and slow-growing neoplasms which, although showing significant chemosensitivity to conventional frontline treatments, are characterized by recurrence. The most widely used frontline chemotherapy scheme is cyclophosphamide, doxorubicin, vincristine, and prednisone (CHOP) associated with cyclophosphamide, doxorubicin, vincristine, and prednisone associated with rituximab (R-CHOP), but rituximab-bendamustine has become an alternative frontline option for many patients.^[1]^ Italian and European guidelines for second-line therapy consist in different options, among which bendamustine can be used as a single agent or in combination with others.^[2,3]^ Here, we describe the case of a 63-year-old patient with fNHL who was switched to rituximab-bendamustine as early second-line treatment after 2 courses of R-CHOP, interrupted because of hematological toxicity and signs of progressive disease. This strategy resulted in complete disease remission, highlighting the effectiveness of the early switch to a bendamustine-based regimen, even during disease progression.

## 2 Case presentation

**Figure 1:**
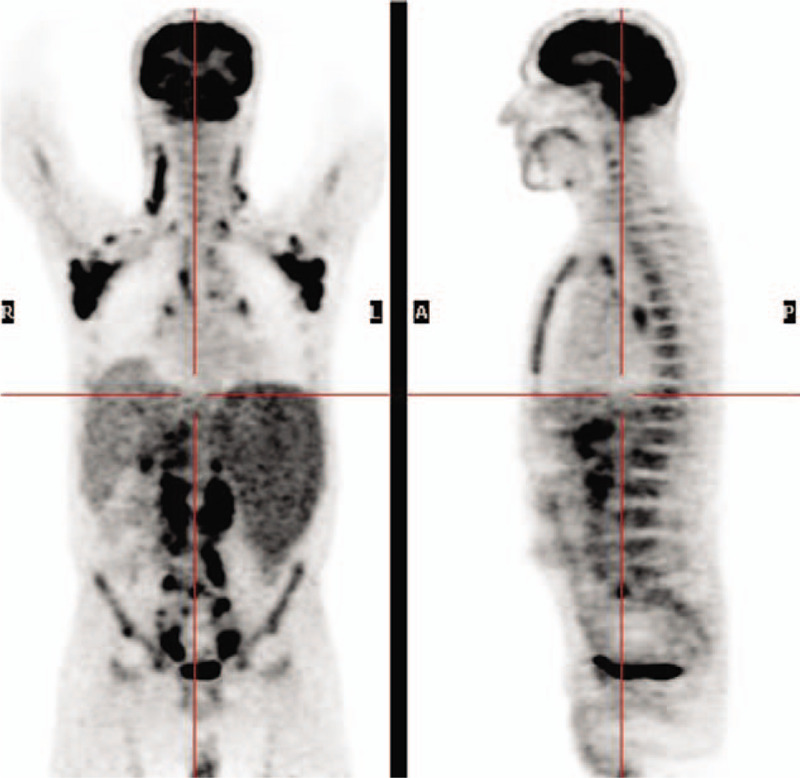
Positron emission tomography/computed tomography scan at diagnosis showed accumulation of fluorodeoxyglucose in several deep and superficial lymph node locations (SUV max range 2–7.7).

**Figure 2:**
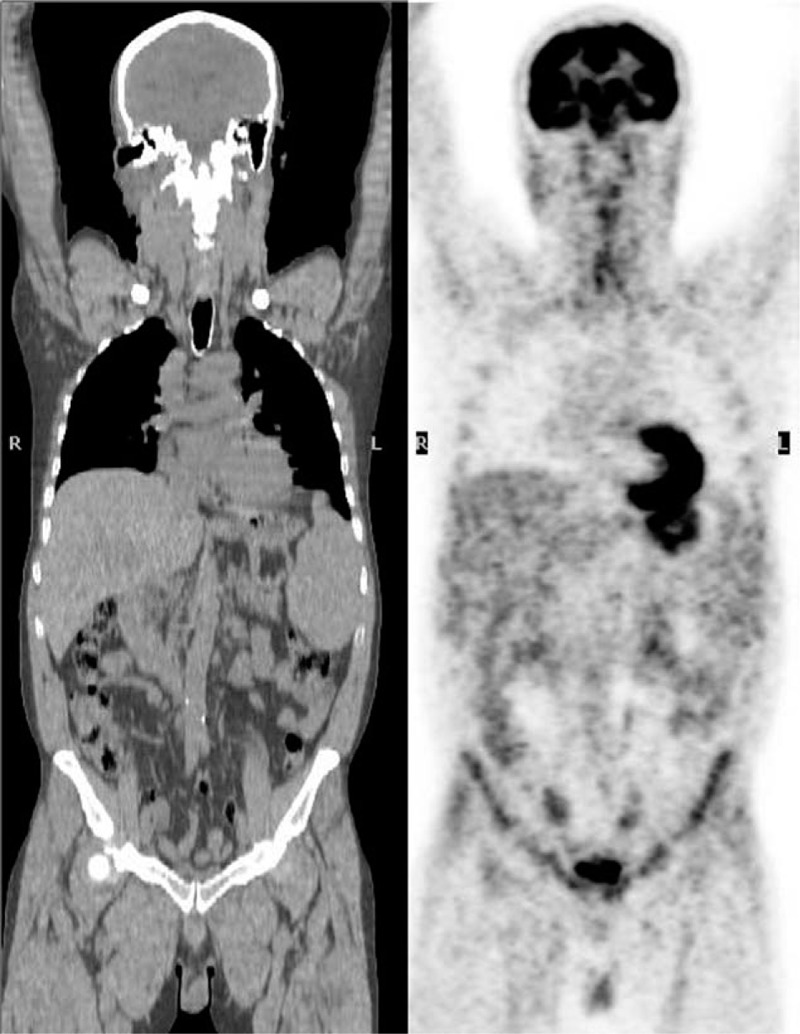
Reevaluation positron emission tomography/computed tomography scan revealed no fluorodeoxyglucose pathological accumulation, confirming clear disease regression.

A 63-year-old Caucasian male was referred to our Hematology Unit because of multiple superficial lymphadenopathies, a 5-kg weight loss in the previous 3 months, anorexia, itching, and copious nocturnal sweating. The patient's general conditions were fairly good and his past medical history was negative. Clinical examination confirmed several lymphadenopathies (up to 3 cm), mild hepatomegaly and notable splenomegaly (20 cm). A complete blood count revealed leukocytosis with lymphocytosis and a blood smear showed small lymphocytes with thickened chromatin and scant cytoplasm. Flow cytometric analysis was positive for CD20, CD10, and CD5. An ^18^F-FDG (fluorodeoxyglucose) PET/CT scan (Fig. 1) showed and accumulation of FDG in several deep and superficial lymph node locations (standardized uptake value (SUV) max range 2–7.7). Excisional biopsy of the largest lymph node in the left axillary region led to a diagnosis of fNHL, stage IV B, CD20+ and bcl-2+. The histological exam showed substantial lymphocytic infiltration (about 82% of the total cell population), while flow cytometry was positive for CD19, CD20, CD10, and k-chains. Conventional frontline treatment with the R-CHOP scheme (rituximab, cyclophosphamide, doxorubicin, vincristine, prednisone) was planned together with supportive therapy consisting in growth factors (pegfilgrastim and erythropoietin β administered from the first cycle onwards). The second course was deferred because of hematological adverse events, that is, common terminology criteria for adverse events grade III anemia and febrile neutropenia, initially treated with ciprofloxacin per os and then substituted with ceftriaxone as fever persisted. After the third cycle of R-CHOP, clinical and instrumental interim reevaluation showed progressive disease. Systemic B-symptoms were still reported by the patients and lymph-nodes were unmodified at clinical evaluation. ^18^F-FDG-PET/CT showed no substantially changes in lymphadenomegaly volumes. The frontline regimen was thus interrupted and switched to bendamustine-rituximab (rituximab 375 mg/m^2^ on day 1 and bendamustine 90 mg/m^2^ on days 1–2) supported by growth factors (pegfilgrastim 6 mg on day 4^[4–9]^ and erythropoietin β 30,000 U/wk, administered from the first cycle onwards), every 28 days for 6 courses. This regimen was well tolerated, patient compliance was optimal, there were no delays in administration and no infectious episodes. An interim reevaluation after three courses revealed that the patient was fit, the blood cell count was normal, and lymphadenopathies and nocturnal sweating had completely regressed. Of note, the ^18^F-FDG PET/CT scan did not show FDG pathological uptake, clearly confirming disease regression (Fig. 2).

## 3 Discussion

R-CHOP is a consolidated chemotherapeutic scheme widely used as frontline treatment for fNHL. There are several second-line options for refractory or relapsed disease among which bendamustine is utilized as a single or combined agent. Our decision to suddenly switch to another chemotherapy regimen was made by clinical findings (no response to frontline treatment), data from literature and, finally, to our clinical experience in mitigate side effects of bendamustine. Response rates with bendamustine in refractory/relapsed patients after a frontline therapy are excellent and well tolerated, with a 90% overall response rate, a complete response rate of up to 69% and progression-free survival of 23 months.^[1,10–14]^

The 2 regimens, R-CHOP and bendamustine-rituximab, were directly compared by Rummel et al in a randomized trial evaluating patients with indolent lymphomas, revealing an advantage for the bendamustine-rituximab scheme in terms of median progression-free survival (69.5 vs 31.2 months, respectively) and tolerability (lower rates of grade 3–4 neutropenia in the former).^[15]^ Another study (BRIGHT trial) confirmed the noninferiority of bendamustine over R-chemotherapy regimens (R-CHOP, R-CVP (Rituximab- C – cyclophosphamide, V – vincristine, P – prednisolone)) in terms of effectiveness as first-line treatment in this setting.^[16]^ Thus, despite the lack of difference in long-term overall survival (OS), bendamustine would seem to be a better choice for patients who are more likely to be susceptible to the toxic effects of more intensive combination treatments such as the elderly or those with numerous comorbidities.

During frontline therapy with R-CHOP, our patient experienced substantial hematological toxicity resulting in severe anemia and neutropenia, even though growth factors were used. In particular, our group has previously demonstrated that primary prophylaxis with pegfilgrastim during bendamustine-containing therapy is more effective than secondary prophylaxis with non-pegylated granulocyte colony-stimulating factors in increasing tolerability and in helping to respect dose-density and dose-intensity, regardless of the type, biology and clinical behavior of the lymphoproliferative disease, for example, lymphoma and multiple myeloma. Severe febrile neutropenia was present, but the short duration and the relatively benign course did not necessitate treatment disruption, allowing us to switch to a more tolerable regimen for the patient.

There is still no clear evidence in the literature of the effectiveness of early treatment switching in patients with indolent lymphomas who relapse or experience severe toxicity whilst undergoing R-CHOP. However, in our patient early second-line treatment with rituximab-bendamustine was well tolerated, obtaining results that make it a potentially effective option for patients with early refractory or relapsed fNHL after frontline therapy, or even for those progressing on conventional chemotherapy. We conclude that early switching to a bendamustine-rituximab-based scheme, even during conventional chemotherapy, decreases toxicity and reduces the risk of treatment interruption or delay, with favorable effects on overall response and prognosis.

## Acknowledgments

The authors thank Gráinne Tierney for language editing.

## Author contributions

Conceptualization, design and writing: Claudio Cerchione, Giovanni Martinelli.

Clinical and research management: Claudio Cerchione, Davide Nappi, Gerardo Musuraca, Alessandro Lucchesi, Ilaria Cimmino, Fabrizio Pane, Amalia De Renzo.

All authors read and approved the final manuscript.

## References

[1] Rummel MJ Al-Batran SE Kim SZ Bendamustine plus rituximab is effective and has a favorable toxicity profile in the treatment of mantle cell and low-grade non-Hodgkin's lymphoma. J Clin Oncol 2005;23:3383–9. 15908650 10.1200/JCO.2005.08.100

[2] Zinzani PL Marchetti M Billio A Expert Panel of the Italian Society of Hematology. SIE, SIES, GITMO revised guidelines for the management of follicular lymphoma. Am J Hematol 2013;88:185–92. 23339086 10.1002/ajh.23372

[3] Dreyling M Ghielmini M Marcus R ESMO Guidelines Working Group. Newly diagnosed and relapsed follicular lymphoma: ESMO Clinical Practice Guidelines for diagnosis, treatment and follow-up. Ann Oncol 2014;25: Suppl 3: 76–82. 10.1093/annonc/mdr388 21908506

[4] Cerchione C De Renzo A Di Perna M Pegfilgrastim in primary prophylaxis of febrile neutropenia following frontline treatment in patients with indolent non-Hodgkin lymphoma: a single center, real-life experience. Support Care Cancer 2017;25:839–45. 27812763 10.1007/s00520-016-3468-8 PMC5266775

[5] Cerchione C Catalano L Pareto AE Pegfilgrastim in primary prophylaxis of febrile neutropenia during chemotherapy of relapsed and refractory multiple myeloma: a real-life experience. Support Care Cancer 2017;23:301–2. 10.1007/s00520-014-2490-y 25341551

[6] Cerchione C Catalano L Peluso I Managing neutropenia by pegfilgrastim in patients affected by relapsed/refractory multiple myeloma treated with bendamustine-bortezomib-dexamethasone. Support Care Cancer 2016;24:4835–7. 27726031 10.1007/s00520-016-3430-9 PMC5082581

[7] Cerchione C De Renzo A Nappi D Pegfilgrastim in primary prophylaxis of febrile neutropenia in elderly patients with hematological malignancies-bendamustine and G-CSF support. Support Care Cancer 2019;27:1587–8. 30671660 10.1007/s00520-019-4651-5

[8] Cerchione C Nappi D Di Perna M A case of efficacy of bendamustine in heavily pretreated multiple myeloma, refractory to pomalidomide. Clin Case Rep 2017;5:505–7. 28396778 10.1002/ccr3.773 PMC5378832

[9] Cerchione C Nappi D Di Perna M Retreatment with bendamustine-bortezomib-dexamethasone in a patient with relapsed/refractory multiple myeloma. Case Rep Hematol 2016;2016:6745286. 27867671 10.1155/2016/6745286 PMC5102715

[10] Cheson BD Friedberg JW Kahl BS Bendamustine produces durable responses with an acceptable safety profile in patients with rituximab-refractory indolent non-Hodgkin lymphoma. Clin Lymphoma Myeloma Leuk 2010;10:452–7. 21189660 10.3816/CLML.2010.n.079

[11] Kahl BS Bartlett NL Leonard JP Bendamustine is effective therapy in patients with rituximab-refractory, indolent B-cell non-Hodgkin lymphoma: results from a Multicenter Study. Cancer 2010;116:106–14. 19890959 10.1002/cncr.24714 PMC2916680

[12] Hiddemann W Kneba M Dreyling M Frontline therapy with rituximab added to the combination of cyclophosphamide, doxorubicin, vincristine, and prednisone (CHOP) significantly improves the outcome for patients with advanced-stage follicular lymphoma compared with therapy with CHOP alone: results of a prospective randomized study of the German Low-Grade Lymphoma Study Group. Blood 2005;106:3725–32. 16123223 10.1182/blood-2005-01-0016

[13] Mondello P Steiner N Willenbacher W Bendamustine plus rituximab versus R-CHOP as first-line treatment for patients with follicular lymphoma Grade 3A: evidence from a multicenter retrospective study. Oncologist 2018;23:454–60. 29317554 10.1634/theoncologist.2017-0037 PMC5896707

[14] Rigacci L Puccini B Cortelazzo S Bendamustine with or without rituximab for the treatment of heavily pretreated non-Hodgkin's lymphoma patients: a multicenter retrospective study on behalf of the Italian Lymphoma Foundation (FIL). Ann Hematol 2012;91:1013–22. 22349722 10.1007/s00277-012-1422-5

[15] Rummel MJ Niederle N Maschmeyer G Bendamustine plus rituximab versus CHOP plus rituximab as first-line treatment for patients with indolent and mantle-cell lymphomas: an open-label, multicentre, randomised, phase 3 non-inferiority trial. Lancet 2013;381:1203–10. 23433739 10.1016/S0140-6736(12)61763-2

[16] Flinn IW van der Jagt R Kahl BS Randomized trial of bendamustine-rituximab or R-CHOP/R-CVP in first-line treatment of indolent NHL or MCL: the BRIGHT study. Blood 2014;123:2944–52. 24591201 10.1182/blood-2013-11-531327 PMC4260975

